# A Novel Fast Iterative STAP Method with a Coprime Sampling Structure

**DOI:** 10.3390/s24124007

**Published:** 2024-06-20

**Authors:** Mingfu Li, Hui Li

**Affiliations:** 1 School of Aeronautics and Astronautics, University of Electronic Science and Technology of China, Chengdu 611731, China; kelly.li@126.com; 2 Chengdu Textile College, Chengdu 611731, China

**Keywords:** space-time adaptive processing, coprime sampling structure, truncated kernel norm, clutter covariance matrix

## Abstract

In space-time adaptive processing (STAP), the coprime sampling structure can obtain better clutter suppression capabilities at a lower hardware cost than the uniform linear sampling structure. However, in practical applications, the performance of the algorithm is often limited by the number of training samples. To solve this problem, this paper proposes a fast iterative coprime STAP algorithm based on truncated kernel norm minimization (TKNM). This method establishes a virtual clutter covariance matrix (CCM), introduces truncated kernel norm regularization technology to ensure the low rank of the CCM, and transforms the non-convex problem into a convex optimization problem. Finally, a fast iterative solution method based on the alternating direction method is presented. The effectiveness and accuracy of the proposed algorithm are verified through simulation experiments.

## 1. Introduction

In airborne radar systems, space-time adaptive processing (STAP) is crucial for clutter suppression and moving target detection [1,2,3,4,5,6,7,8,9,10,11,12,13]. However, the performance of the STAP algorithm is largely affected by the accuracy of clutter covariance matrix (CCM) estimation. In practical applications, radar systems face complex clutter environments generated by factors such as terrain, buildings, and weather conditions. These clutters not only vary significantly in intensity but also possess distinct spatial and temporal characteristics. The received radar signal can be expressed as x=s+c+n, where s represents the target echo signal, c represents the clutter signal, and n represents the noise signal. The goal of the STAP algorithm is to extract the target echo signal s from the received signal x while suppressing the clutter signal c and noise signal n. The clutter covariance matrix is a crucial parameter that describes the statistical properties of the clutter signal, including its spatial and temporal correlations. Ideally, the clutter covariance matrix can be estimated using independently and identically distributed training samples from adjacent cells (without targets). Typically, the CCM is estimated using independent and identically distributed training samples (without targets) of the neighboring test cells. But, in a heterogeneous environment, it is difficult to meet this requirement. In addition, in order to ensure that the signal-to-interference-to-noise ratio (SINR) loss does not exceed 3 dB, the number of training samples should be at least twice the system degrees of freedom (DOF) [14]. However, the full vector filters require significant computational complexity and storage space. Therefore, algorithms with low samples and low computational complexity are particularly important in practical applications.

For decades, researchers have proposed various algorithms to estimate the CCM with limited training samples. These algorithms include reduced dimension [14,15], reduction-rank methods [16,17,18,19], and persymmetry methods [20,21], which can reduce the need for training samples. In addition, the direct data domain method [22] only uses test data and avoids dependence on training samples. However, these methods usually sacrifice system DOF, resulting in performance degradation. In recent years, the emergence of sparse arrays has provided new ideas for solving these problems. Through difference operations, sparse arrays can obtain virtual arrays similar to physical arrays, allowing the system to have more virtual sensors than physical sensors. In addition, the larger element spacing in the sparse array also significantly reduces the mutual coupling effect of the array, further improving the performance and stability of the system.

Common sparse arrays include minimal redundancy arrays [23,24], nested arrays [5,25,26,27], and coprime arrays [5,28,29,30,31]. Minimally redundant arrays provide the largest contiguous array for a given number of sensors, but lack closed expressions for sensor locations and DOF. Compared with nested arrays, coprime arrays have relatively weak mutual couple effects due to the large spacing between array sensors. However, a large number of holes appear in the difference coarray, which affects the full utilization of virtual elements. Due to the low rank of the CCM, it can be estimated by the compressed sensing algorithm. The literature [32] uses the symmetric structure [33] and its positive definiteness [34] of the covariance matrix to construct the kernel norm and proposes a sparse sensing algorithm that uses the cyclic rank minimization method to reconstruct the covariance matrix. Meanwhile, the literature [35] proposed an array interpolation algorithm using atomic norm minimization. However, the research results in [36] show that the rank kernel norm relaxation cannot guarantee the low rank of the CCM of the multi-level Toplitz matrix. In [37], Cai et al. demonstrated that kernel norm minimization based on low-rank matrix estimation problems can be solved through singular value contraction operations. In addition, Lin et al. [38] proposed an exact iterative method called augmented Lagrange multiplier to solve this problem. Although these algorithms perform well in terms of estimation accuracy, a large number of sensors are required to achieve the performance of these algorithms in practical applications. It has relatively high computational complexity, which poses challenges to the real-time performance and efficiency of the system. In order to overcome these problems, this paper proposes a fast iterative coprime STAP algorithm (TNNM-FIC-STAP) based on truncated kernel norm minimization.

First, a detailed analysis of the coprime sampling structure of the STAP is presented, along with the corresponding CCM determined in the virtual domain. Then, the concept of truncated kernel norm is introduced, and a fast iterative coprime STAP algorithm based on this theory is proposed. The final solution comes from the convex relaxation process. Finally, the effectiveness of the algorithm is verified through numerous simulation experiments.

The content of this article is arranged as follows: Section 2 introduces the coprime sampling structure model, difference operation, and filter weight vector estimation process. In Section 3, the truncated nuclear norm and its fast iteration process are explained. In Section 4, simulation results are used to verify the performance advantages of the algorithm. Finally, Section 5 summarizes the main contents of this article and presents conclusions. The list of abbreviations used in the text is presented in Table 1.

## 2. Coprime Sampling Structured STAP

### 2.1. Coprime Sampling Structured Model

**Figure 1:**
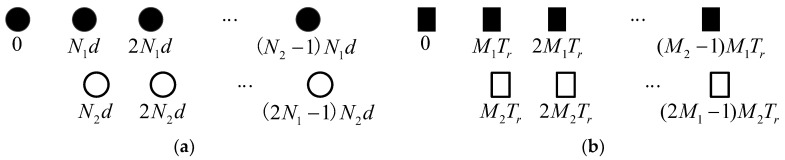
Coprime configuration: (**a**) coprime array; (**b**) coprime PRI.

Assuming a side-looking airborne phased array radar with a coprime sampling structure for both the array and pulse repetition interval (PRI), which emits M pulses in a coherent processing interval (CPI) and has N array sensors, as shown in Figure 1. The array consists of two sub-uniform linear arrays (ULAs). The denser sub-ULA contains $N_{2}$ array elements at position $\left\{N_{1}p_{2}d,0\leq p_{2}\leq N_{2}-1\right\}$, the sparser sub-ULA contains $2N_{1}-1$ array sensors at position $\left\{N_{2}p_{1}d,1\leq p_{1}\leq 2N_{1}-1\right\}$, where $N_{1}$ and $N_{2}$ are mutually prime integers satisfying $N_{1}<N_{2}$, d is the minimum distance between array sensors, as shown in Figure 1a. Similar to the coprime array structure, Figure 1b illustrates the coprime PRI structure. The impulse consists of two uniform sub-impulse, containing $M_{2}$ and $2M_{1}-1$ impulses, respectively. The position coordinates of the sub-impulses are $\left\{M_{1}q_{2}T_{r},0\leq q_{2}\leq M_{2}-1\right\}$ and $\left\{M_{2}q_{1}T_{r},1\leq q_{1}\leq 2M_{1}-1\right\}$, where $M_{1}$ and $M_{2}$ are mutually prime integers satisfying $M_{1}<M_{2}$. The minimum PRI is $T_{r}$. Without the ranger ambiguity, the space-time snapshot received from a range bin can be expressed as

(1) $$x=a_{t}v(\varphi_{t},f_{t})+x_{u}$$

where $a_{t}$ represents the target complex amplitude, and $v(\varphi_{t},f_{t})=v(\varphi_{t})⊗v(f_{t})$ is the target space-time steering vector. And the spatial steering vector is

(2) $$v(\varphi_{t})={\left[1,e^{2\pi jn_{1}\varphi_{t}},\ldots ,e^{2\pi jn_{N-1}\varphi_{t}}\right]}^{T}$$

and the time steering vector is

(3) $$v(f_{t})={\left[1,e^{2\pi jm_{1}f_{t}},\ldots ,e^{2\pi jm_{M-1}f_{t}}\right]}^{T}$$

where $\varphi_{t}=d\cos(\theta )/\lambda$, $f_{t}=2v_{r}T_{r}\cos(\theta )/\lambda$, λ is the radar’s wavelength, $v_{r}$ is the radar velocity, and θ is the spatial cone angle where the target is located. Assuming that $N_{c}$ is the number of independent clutter patches representing in a range ring, then the clutter plus noise data $x_{u}$ is

(4) $$x_{u}=\sum_{i=1}^{N_{c}}a_{c,i}v(\varphi_{c,i},f_{c,i})+n=\sum_{i=1}^{N_{c}}a_{c,i}v(\varphi_{c,i})⊗v(f_{c,i})+n$$

The normalized spatial and time frequencies of the ith clutter patch are $\varphi_{c,i}$ and $f_{c,i}$, respectively. $a_{c,i}$ is its complex amplitude, n is the noise vector, and the space steering vector is

(5) $$v(\varphi_{c,i})={\left[1,e^{2\pi jn_{1}\varphi_{c,i}},\ldots ,e^{2\pi jn_{N-1}\varphi_{c,i}}\right]}^{T}.$$

The time steering vector is

(6) $$v(f_{c,i})={\left[1,e^{2\pi jm_{1}f_{c,i}},\ldots ,e^{2\pi jm_{M-1}f_{c,i}}\right]}^{T}$$

and the corresponding space-time steering vector is

(7) $$v(\varphi_{c,i},f_{c,i})=v(\varphi_{c,i})⊗v(f_{c,i})=\left[\begin{array}{c} 1\\ e^{2\pi jn_{1}\varphi_{c,i}}\\ \vdots \\ e^{2\pi jn_{N-1}\varphi_{c,i}} \end{array}\right]⊗\left[\begin{array}{c} 1\\ e^{2\pi jm_{1}f_{c,i}}\\ \vdots \\ e^{2\pi jm_{M-1}f_{c,i}} \end{array}\right]=\left[\begin{array}{c} v_{0,i}\\ v_{1,i}\\ \vdots \\ v_{NM-1,i} \end{array}\right]$$

where $v_{lM+r-1,i}=e^{2\pi j(n_{l}\varphi_{c,i}+m_{r-1}f_{c,i})}$, l=0,⋯,N−1, r=1,⋯,M, $i=1,\cdots ,N_{c}$. From formula (4), the clutter plus noise covariance matrix (CNCM) $R_{u}$ can be calculated by

(8) $$\begin{array}{cc} R_{u} & =E\left[x_{u}{x_{u}}^{H}\right]\\ & =\sum_{i=1}^{N_{c}}E({\left|a_{c,i}\right|}^{2})v(\varphi_{c,i},f_{c,i})v^{H}(\varphi_{c,i},f_{c,i})+Q_{NM}\\ & =VPV^{H}+Q_{NM}\\ & =R_{c}+Q_{NM}, \end{array}$$

where $V=[v(f_{c,1},\varphi_{c,1}),v(f_{c,2},\varphi_{c,2}),\ldots ,v(f_{c,N_{c}},\varphi_{c,N_{c}})]$ is the clutter space-time steering matrix, $P=diag({\left[p_{1},p_{2},\ldots ,p_{N_{c}}\right]}^{T}),\;p_{k}=E({\left|a_{c,k}\right|}^{2})$ represents the clutter power matrix, the CCM can be expressed as $R_{c}$, and $Q_{NM}=diag(\sigma_{1}^{2},\sigma_{2}^{2},\ldots ,\sigma_{MN}^{2})$ is the noise covariance matrix. The background noise is the Gaussian white noise when the ideal condition $Q_{NM}=\sigma_{n}^{2}I_{MN}$ is satisfied.

### 2.2. Difference Operation

**Figure 2:**
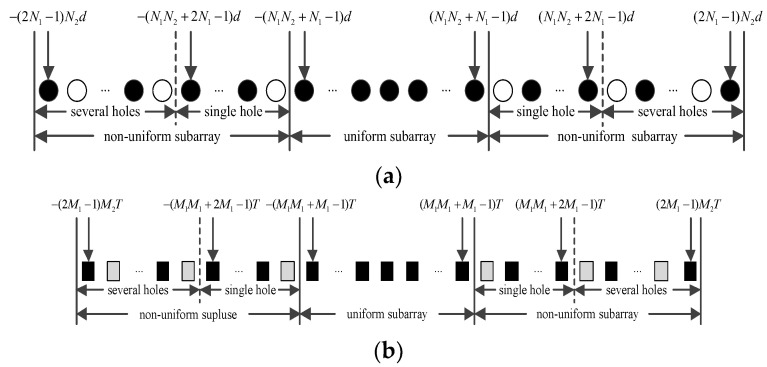
Difference coarray and copulse: (**a**) difference array; (**b**) difference impulses.

To clearly explain the virtual array and impulse train, define the difference coarray D of the array A as

(9) $$D=\{d_{n1}-d_{n2}|d_{n1},d_{n2}\in A\}$$

where $d_{n1}$ is the position of the n1 array sensor. As shown in Figure 2a, the de-duplication set $D_{l}$ from set D is

(10) $$D_{l}=\{-(2N_{1}-1)N_{2},-(2N_{1}-2)N_{2},\cdot \cdot \cdot ,(2N_{1}-1)N_{2}\}.$$

Holes in the arrays appear after $-(N_{1}N_{2}+N_{1}-1)$ and $\left(N_{1}N_{2}+N_{1}-1\right)$, and two discontinuous difference sub-arrays are symmetric. For a sparse impulse train ℙ, the difference operations set ℚ is

(11) $$\mathbb{Q}=\{q_{m1}-q_{m2}|q_{m1},q_{m2}\in \mathbb{P}\}$$

with its de-duplication set ${\mathbb{Q}}_{l}$

(12) $${\mathbb{Q}}_{l}=\{-(2M_{1}-1)M_{2},-(2M_{1}-2)M_{2},\cdot \cdot \cdot ,(2M_{1}-1)M_{2}\}$$

As shown in Figure 2b, the structure and properties of impulses are similar to those of different arrays. To enlarge the DOF in the space and time domains, virtual array sensors and impulses can be increased by filling the holes.

A filled circle stands for physical sensors and an empty circle represents empty space. A filled rectangle stands for transmitting pulses and an empty rectangle represents empty space.

### 2.3. The STAP Method

Equation (8) can be written as

(13) $$\begin{array}{l} R_{u}=R_{c}+Q_{NM}\\ \;=\left[\begin{array}{c} R_{0,0}\\ R_{1,0}\\ \vdots \\ R_{NM-1,0} \end{array}\begin{array}{c} R_{0,1}\\ R_{1,1}\\ \vdots \\ R_{NM-1,1} \end{array}\begin{array}{c} \cdots \\ \cdots \\ \ddots \\ \cdots \end{array}\begin{array}{c} R_{0,NM-1}\\ R_{1,NM-1}\\ \vdots \\ R_{NM-1,NM-1} \end{array}\right]+\left[\begin{array}{c} \sigma_{1}^{2}\\ 0\\ \vdots \\ 0 \end{array}\begin{array}{c} 0\\ \sigma_{2}^{2}\\ \vdots \\ 0 \end{array}\begin{array}{c} \cdots \\ \cdots \\ \ddots \\ \cdots \end{array}\begin{array}{c} 0\\ 0\\ \vdots \\ \sigma_{MN}^{2} \end{array}\right] \end{array}$$

where

(14) $$R_{\left(l_{1}M+r_{1}-1),(l_{2}M+r_{2}-1\right)}=\sum_{k=1}^{N_{c}}p_{k}e^{2\pi j[(n_{l_{1}}-n_{l_{2}})\varphi_{c,k}+(m_{r_{1}-1}-m_{r_{2}-1})f_{c,k}]}$$

and $l_{1},l_{2}=0,\cdots ,N-1,\;r_{1},r_{2}=1,\cdots ,M$.

By comparing Equations (10), (12) and (13), it can be seen that the elements in the CCM correspond to the virtual sensors and the virtual impulse positions. To downplay the effects of the background noise on the covariance matrix elements, the covariance matrix elements corresponding to the same virtual array sensor and the virtual pulse are averaged. Let the ith element in $R_{u}$ corresponding to the $d_{n}$ virtual sensor and the $q_{m}$ virtual pulse be $R_{u}^{i}(d_{n},q_{m})$, then the mean value is

(15) $${\bar{R}}_{u}(d_{n},q_{m})=\frac{1}{w(d_{n},q_{m})}\sum_{i=1}^{w(d_{n},q_{m})}R_{u}^{i}(d_{n},q_{m})$$

where $w(d_{n},q_{m})$ denotes the number of times the array difference $d_{n}$ and impulse difference $q_{m}$ are repeated at the same time. Now, $R_{u}$ is

(16) $$R_{u}=R_{1}+R_{2}\;$$

where

(17) $${\left[R_{1}\right]}_{i,j}=\left\{ \begin{array}{ll} {\left[R_{u}\right]}_{i,j}, & i\neq j\\ 0, & i=j \end{array}\right.$$

(18) $$R_{2}=diag({\left[R_{u}\right]}_{1,1},{\left[R_{u}\right]}_{2,2},\ldots ,{\left[R_{u}\right]}_{MN,MN})$$

and ${\left[R_{u}\right]}_{i,i}=R_{i,i}+\sigma_{i}^{2}$.

To reduce the impact of non-uniform noise on the covariance matrix, all $\sigma_{i}^{2}$ are replaced with $\sigma_{\min}^{2}=\mathrm{MIN}(\sigma_{i}^{2})$ to whiten it. In this way, the influence of non-uniform noise can be suppressed and the signal-to-noise ratio (SNR) can be improved. At this time, the estimated value of $R_{2}$ is denoted as ${\hat{R}}_{2}$, with its corresponding covariance matrix denoted as ${\hat{R}}_{u}$.

To increase the number of virtual array sensors and impulses, all holes are filled in the difference coarray, and the numbers of virtual sensors and virtual impulses are now $4N_{1}N_{2}-2N_{2}+1$ and $4M_{1}M_{2}-2M_{2}+1$, respectively. Extending the covariance matrix ${\hat{R}}_{u}$ with zero padding results in an $\left(4N_{1}N_{2}-2N_{2}+1)\times (4M_{1}M_{2}-2M_{2}+1\right)$ matrix $R_{E}$ by the existing elements of wave range difference and impulse difference. It is assumed that $R_{E}=R_{1E}+R_{2E}$, where $R_{1E}$ and $R_{2E}$ are the corresponding recovery matrices for $R_{1}$ and $R_{2}\;$, respectively.

## 3. Fast Iterative Coprime STAP Method

### 3.1. Truncated Kernel Norm

The truncated kernel norm of the matrix $R\in {\mathbb{R}}^{m\times n}(m\geq n)$ is defined as the sum of the smaller n−r singular values. Namely,

(19) $${\left\|R\right\|}_{r}=\sum_{i=1}^{n}\sigma_{i}-\sum_{i=1}^{r}\sigma_{i}=\sum_{i=r+1}^{n}\sigma_{i}$$

where $\sigma_{i}$ is the ith singular value. The singular value decomposition (SVD) of the matrix R is $R=U\Sigma V^{T}$, $U=\left[u_{1},u_{2},\cdots u_{m}\right]$, $V=\left[v_{1},v_{2},\cdots v_{n}\right]$, $\Sigma =\mathrm{diag}(\sigma_{1},\sigma_{2},\cdots ,\sigma_{n})$. For $\forall R\in {\mathbb{R}}^{m\times n}(m\geq n)$ and ∀r<n, we have

(20) $$tr(XRY^{T})\leq \sum_{i=1}^{r}\sigma_{i},r\in N.$$

Here, $\forall X\in {\mathbb{R}}^{r\times m}$ and $\forall Y\in {\mathbb{R}}^{r\times n}$ satisfy $XX^{T}=I,\;YY^{T}=I$. When $X={\left[u_{1},u_{2},\cdots u_{r}\right]}^{T}=U_{r}^{T}$ and $Y={\left[v_{1},v_{2},\cdots v_{r}\right]}^{T}=V_{r}^{T}$, the maximum value of $tr(XRY^{T})$ is obtained by

(21) $$\max\;tr(XRY^{T})=tr(U_{r}^{T}RV_{r})=\sum_{i=1}^{r}\sigma_{i}.$$

### 3.2. TNNM-STAP Method

From Equation (19), we have

(22) $$\underset{R_{1E},R_{2E}\;}{\min}{\left\|R_{1E}\right\|}_{r}+\lambda {\left\|R_{2E}\;\right\|}_{1},\;s.t.\;R_{E}=R_{1E}+R_{2E}$$

where ${\left\|R_{1E}\right\|}_{r}$ is non-convex that cannot be directly solved, but it can be transformed into [39].

(23) $$\underset{R_{1E},R_{2E}\;}{\min}{\left\|R_{1E}\right\|}_{*}-\underset{XX^{T}=I,YY^{T}=I}{\max}tr(XR_{1E}Y^{T})+\lambda {\left\|R_{2E}\;\right\|}_{1},\;s.t.\;R_{E}=R_{1E}+R_{2E}$$

The first r larger singular values are assigned a weight of 0 and the remaining n-r smaller ones are assigned a weight of 1. This preserves the main component of the data and removes the noise. The $\mathrm{tr}(XR_{1E}Y^{T})$ reaches its maximum value when $X=U_{r}^{T}$ and $Y=V_{r}^{T}$, so its low-rank matrix can be recovered in two steps. In the kth iteration, the left singular vectors $U_{r}$ and right singular vectors $V_{r}$ obtained by singular value decomposition of $R_{1Ek}$ can optimize Equation (22) to

(24) $$\underset{R_{1E},R_{2E}\;}{\min}{\left\|R_{1E}\right\|}_{*}-tr(U_{r}^{T}R_{1E}V_{r})+\lambda {\left\|R_{2E}\;\right\|}_{1},\;s.t.\;R_{E}=R_{1E}+R_{2E}.$$

The two steps are executed alternately until convergence. The specific steps are

**Input:** original matrix $R_{E}$, termination error ε, and $R_{0}=0$. 

**Output:** ${\hat{R}}_{1E},\;{\hat{R}}_{2E}$.

**Step 1:** singular value decomposition of $R_{1Ek}$:

$$\left[U_{k},\sum_{k},V_{k}\right]=\mathrm{SVD}(R_{1Ek})\;U_{r}=\left[u_{1},u_{2},\cdots u_{r}\right]\in {\mathbb{R}}^{m\times r},\;V_{r}=\left[v_{1},v_{2},\cdots v_{r}\right]\in {\mathbb{R}}^{m\times r}.$$

**Step 2:** solve the optimization problem:

$$(R_{1E(k+1)},R_{2E(k+1)})=\arg\underset{R_{1E},R_{2E}\;}{\min}{\left\|R_{1E}\right\|}_{*}-tr(U_{r}^{T}R_{1E}V_{r})+\lambda {\left\|R_{2E}\;\right\|}_{1},\;s.t.\;R_{E}=R_{1E}+R_{2E}.$$

Repeat steps 1 and 2 for iterative operations. 

When ${\left\|R_{E}-R_{1E(k+1)}-R_{2E(k+1)}\right\|}_{F}/{\left\|R_{E}\right\|}_{F}\leq \varepsilon$, stop iteration and return to ${\hat{R}}_{1E},\;{\hat{R}}_{2E}$.

Here, the alternating direction method is used to update $R_{1Ek}$ and $R_{2Ek}$ to solve Equation (24). The corresponding augmented Lagrangian function can be written as

(25) $$\begin{array}{ll} L(R_{1E},R_{2E},Y,\mu ) & ={\left\|R_{1E}\right\|}_{*}-tr(U_{r}^{T}R_{1E}V_{r})+\lambda {\left\|R_{2E}\;\right\|}_{1}\\ & =+tr(Y^{T}(R_{E}-R_{1E}-R_{2E}))+\frac{\mu }{2}{\left\|R_{E}-R_{1E}-R_{2E}\;\right\|}_{F}^{2} \end{array}$$

where the Lagrange multiplier is Y, and the penalty coefficient is μ. The solution steps for the kth iteration are as follows:

(1) Given $R_{1Ek}$, $Y_{k}$, and $\mu_{k}$, we have
  (26) $$\begin{array}{l} R_{2E(k+1)}=\arg\underset{R_{2E}\;}{\min}L(R_{1Ek},R_{2E},Y_{k},\mu_{k})\\ =\arg\underset{R_{2E}\;}{\min}\{\lambda {\left\|R_{2E}\;\right\|}_{1}+tr(Y_{k}^{T}(R_{E}-R_{1E}-R_{2E}))+\frac{\mu_{k}}{2}{\left\|R_{E}-R_{1Ek}-R_{2E}\;\right\|}_{F}^{2}\}\\ =\arg\underset{R_{2E}\;}{\min}\{\lambda {\left\|R_{2E}\;\right\|}_{1}+\frac{\mu_{k}}{2}{\left\|R_{2E}-(R_{E}-R_{1Ek}+\mu_{k}^{-1}Y_{k})\;\right\|}_{F}^{2}\} \end{array}.$$

According to the soft-thresholding method [40], we can know that

(27) $$R_{2E(k+1)}=S_{\lambda \mu_{k}^{-1}}(R_{E}-R_{1Ek}+\mu_{k}^{-1}Y_{k}),S_{\delta }(Z)=\max(\left\|z_{i,j}\right\|-\delta ,0)\frac{z_{i,j}}{{\left\|z_{i,j}\right\|}_{2}}$$

where $z_{i,j}$ is the element of the ith row and jth column of the matrix $Z=R_{E}-R_{1Ek}+\mu_{k}^{-1}Y$, $\delta =\lambda \mu_{k}^{-1}$.

(2) Given $R_{2Ek}$, $Y_{k}$, and $\mu_{k}$, we have
  (28) $$\begin{array}{l} R_{1E(k+1)}=\arg\underset{R_{1E}\;}{\min}L(R_{1E},R_{2E(k+1)},Y_{k},\mu_{k})\\ =\arg\underset{R_{2E}\;}{\min}\{\begin{array}{l} {\left\|R_{1E}\;\right\|}_{*}-tr({\left(U_{r}^{T}\right)}_{k}R_{1E}{\left(V_{r}\right)}_{k})\\ +tr(Y_{k}^{T}(R_{E}-R_{1E}-R_{2E(k+1)}))+\frac{\mu_{k}}{2}{\left\|R_{E}-R_{1E}-R_{2E(k+1)}\;\right\|}_{F}^{2} \end{array}\} \end{array}$$
  which is equivalent to
  (29) $$R_{1E(k+1)}=\arg\underset{R_{2E}\;}{\min}\{{\left\|R_{1E}\;\right\|}_{*}+\frac{\mu_{k}}{2}{\left\|\begin{array}{l} R_{1E}-\\ \left[\begin{array}{l} R_{E}-R_{2E(k+1)}\\ +\mu_{k}^{-1}(Y_{k}+{\left(U_{r}\right)}_{k}{\left(V_{r}^{T}\right)}_{k}) \end{array}\right]\; \end{array}\right\|}_{F}^{2}\}.$$

According to the Singular Value Threshold (SVT) method [37], the above problem turns into

(30) $$R_{1E(k+1)}={ℑ}_{\mu_{k}^{-1}}(R_{E}-R_{2E(k+1)}+\mu_{k}^{-1}(Y_{k}+{\left(U_{r}\right)}_{k}{\left(V_{r}^{T}\right)}_{k}))=US_{\mu_{k}^{-1}}(\Sigma )V^{T}$$

(31) $$S_{\mu_{k}^{-1}}(\Sigma )=diag\{\max\left[(\sigma_{i}-\mu_{k}^{-1}),0\right]\}$$

where vectors U and V are the left and right singular vectors of matrix $Q=R_{E}-R_{2E(k+1)}+\mu_{k}^{-1}(Y_{k}+{\left(U_{r}\right)}_{k}{\left(V_{r}^{T}\right)}_{k})$, and $\sigma_{i}$ being the ith singular value.

(3) Given $R_{1E(k+1)}$, $R_{2E(k+1)}$, and $\mu_{k}$, we have
  (32) $$Y_{\left(k+1\right)}=Y_{k}+\mu_{k}\left(R_{E}-R_{1E(k+1)}-R_{2E(k+1)}\right).$$
(4) Given $R_{1E(k+1)}$, $R_{2E(k+1)}$, and $Y_{\left(k+1\right)}$, we have
  (33) $$\mu_{k+1}=\min(\mu_{k}\rho ,\mu_{\max})$$
  where ρ>1 is a constant. The larger μ is, the faster the algorithm converges.

The above iterative algorithm updates $\{R_{1E(k+1)},R_{2E(k+1)}\}$, which in turn updates $\{U_{r},V_{r}\}$ in step 1, until the iteration stops. The rank r of the reconstructed matrix is needed in the above process, but in practice, it is usually impossible to predict. If r is set too small, there would be reduced low-rank parts and inaccurate reconstruction. And if r is set too large, there would be slow convergence and poor timeliness. To reduce the rank dependence, the rank estimation is performed by the soft-thresholding method. Meanwhile, the SVD operation is replaced by the fast iteration to reduce the algorithm’s computational workload and complexity.

By introducing a soft thresholding operator into the calculation of singular values in Equation (30), Equation (31) is abbreviated as

(34) $$S_{\mu_{k}^{-1}}(\Sigma_{i,j})=\left\{ \begin{array}{ll} \sigma_{i}-\mu_{k}^{-1}, & if\;\sigma_{i}>\mu_{k}^{-1}\\ 0, & \mathrm{otherwise} \end{array}\right.$$

Now, $R_{1E(k+1)}=US_{\mu_{k}^{-1}}(\Sigma_{i,j})V^{T}$. The above equation tells that the value of non-zero elements in $S_{\mu_{k}^{-1}}(\Sigma_{i,j})$ come by threshold $\;\mu_{k}^{-1}$, where the rank of the estimated matrix is the number of the non-zero elements, denoted as $\hat{r}$. So, the algorithm steps become

**Input:** original matrix $R_{E}$ and termination error ε. $R_{0}=0$, $Y_{0}=D/J(D)$, $J(D)=\max({\left\|D\right\|}_{2},\lambda^{-1}{\left\|D\right\|}_{\infty })$, ${\left(U_{r}\right)}_{0}=0$, ${\left(V_{r}\right)}_{0}=0$, $r_{0}=0$.

**Output:** ${\hat{R}}_{1E},{\hat{R}}_{2E}$. 

**Step 1**: $R_{2E(k+1)}=S_{\lambda \mu_{k}^{-1}}\left(R_{E}-R_{1Ek}+\mu_{k}^{-1}Y_{k}\right)$.

**Step 2:** $R_{1E(k+1)}={ℑ}_{\mu_{k}^{-1}}\left(R_{E}-R_{2E(k+1)}+\mu_{k}^{-1}(Y_{k}+{\left(U_{r}\right)}_{k}{\left(V_{r}^{T}\right)}_{k})\right)$.

**Step 3:** sum ${\left(U_{r}\right)}_{k+1}$ and ${\left(V_{r}\right)}_{k+1}$, and estimate the rank $r_{k+1}$.

**Step 4:** $Y_{\left(k+1\right)}=Y_{k}+\mu_{k}\left(R_{E}-R_{1E(k+1)}-R_{2E(k+1)}\right)$.

**Step 5:** $\mu_{k+1}=\min(\mu_{k}\rho ,\mu_{\max})$.

Repeat steps 1–5, until ${\left\|R_{E}-R_{1Ek}-R_{2Ek}\right\|}_{F}/{\left\|R_{E}\right\|}_{F}\leq \varepsilon$.

The penalty parameter μ is automatically updated to increase the number of iterations and speed up convergence. A constant ρ greater than 1 must be chosen appropriately since a value too large would cause the algorithm to diverge. To demonstrate the speed of the proposed algorithm compared to ordinary iterative algorithms, Table 2 provides the specific complexity of each step of the algorithm.

## 4. Simulation Experiments

This section verifies the performance of the TNNM-FIC-STAP by comparing it with traditional STAP (T-STAP) and traditional coprime STAP (C-STAP) in terms of root mean square error (RMSE), system DOF, Beampatterns, and SCNR. The main parameters of the airborne radar system are listed in Table 3, and β=1. We set $N_{1}=M_{1}=2$, $N_{2}=M_{2}=3$ ρ=1.5, $\mu_{\max}=10^{10}$, $\mu_{0}={1.25/\left\|R_{E}\right\|}_{2}$. All simulation results are averaged over 500 Monte Carlo experiments.

### 4.1. The Root Mean Square Error (RMSE)

The root mean square error (RMSE) of matrix estimation is defined as

(35) $$\mathrm{RMSE}=\sqrt{\frac{E\{{\left\|{\hat{R}}_{c}-R_{c}\right\|}^{2}\}}{{\left\|R_{c}\right\|}^{2}}}$$

**Figure 3:**
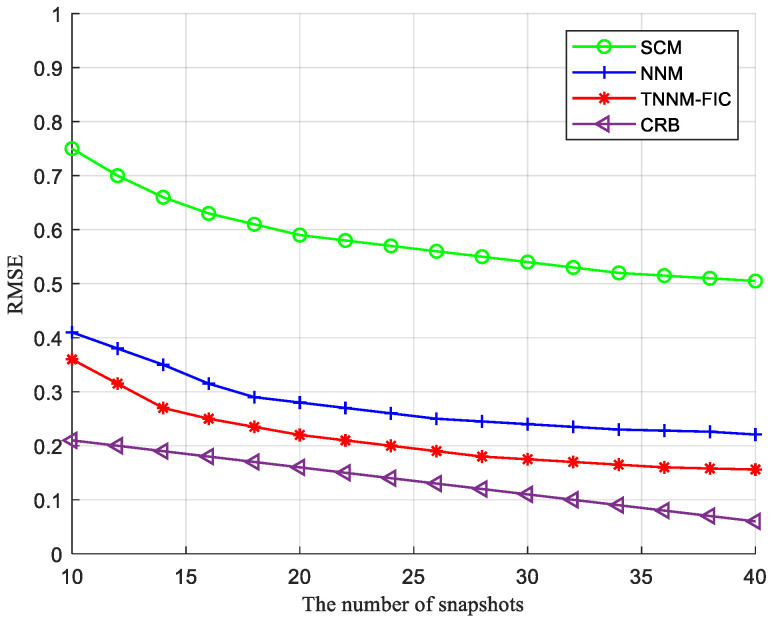
Relationship of the RMSE and the number of samples.

Figure 3 shows the relationships between the RMSE and the number of samples for the sample covariance matrix (SCM) method, the minimum nuclear norm (NNM) method, and the proposed method (TNNM-FIC).

It shows that the performance of NNM and TNNM-FIC is superior to that of SCM. The reconstruction errors of TNNM-FIC and NNM are also relatively stable. The overall relative error of TNNM-FIC reconstruction decreases rapidly, and the convergence speed is much faster than NNM and SCM. The convergence speed of the NNM is also fast, and as the number of iterations increases, the overall relative error of the NNM remains basically unchanged, while the TNNM-FIC is still decreasing. Although the overall relative error of the NNM can be achieved, it requires a larger sample size.

### 4.2. DOF

Compare the performance of the three algorithms from the system DOF. The ratio of DOF is defined as

(36) $$\gamma (K)=L(K)/L_{TNNM-FIC}(K)$$

where $L_{TNNM-FIC}$ is the maximum DOF of the TNNM-FIC-STAP when N=M=K.

**Figure 4:**
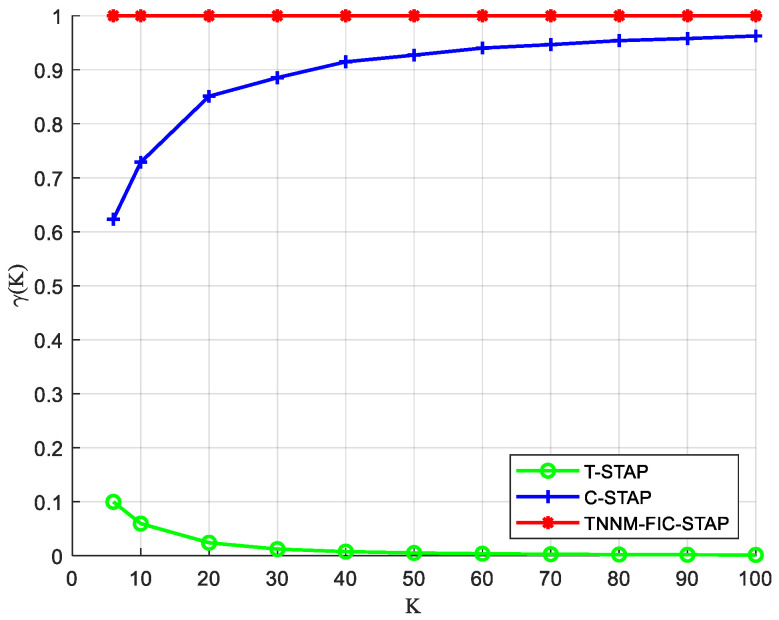
The ratio of DOF.

It can be seen from Equation (36) that the size of DOF is proportional to γ(K). As shown in Figure 4, the C-STAP acquires a larger DOF in the virtual domain than the T-STAP due to the different structure. The TNNM-FIC-STAP uses the low-rank characteristics of the matrix to fill its holes, fully utilizes the virtual array sensors and virtual pulses in the difference structure, and its system DOF is relatively maximized. When K is any value, the DOF of the TNNM-FIC-STAP is higher than that of the other two algorithms. Especially when the value of K is small, the superiority of the TNNM-FIC-STAP is most significant. As its value grows, the DOF of the C-STAP becomes closer and closer to it until it is the same. However, the TNNM-FIC-STAP is more complex than the C-STAP algorithm, so its value must be selected reasonably in practical applications.

### 4.3. Beampatterns

**Figure 5:**
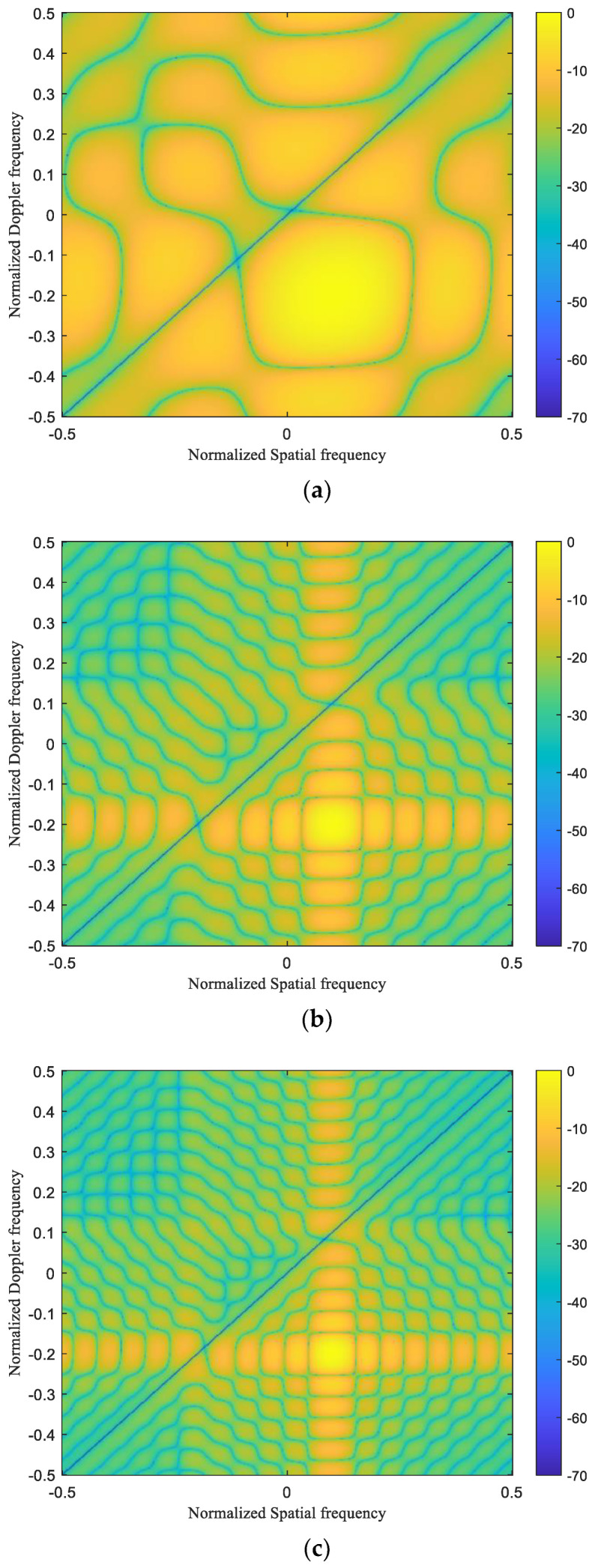
Beampatterns in angle Doppler: (**a**) T-STAP; (**b**) C-STAP; (**c**) TNNM-FIC-STAP.

Figure 5 shows the space-time Beampatterns of T-STAP, C-STAP and TNNM-FIC-STAP. The optimal Beampatterns are formed at the target location (normalized spatial frequency of 0.1 and normalized temporal frequency of −0.2) for the three algorithms. However, the TNNM-FIC-STAP shows a narrow main beam and lower resolution than the other two algorithms in the Doppler and spatial Beampatterns. Of all the three algorithms, there are notches as deep as 30 dB on the main clutter ridges, which can be suppressed effectively. As the system DOF increases, the notches become deeper, and the main clutter is easier to suppress.

**Figure 6:**
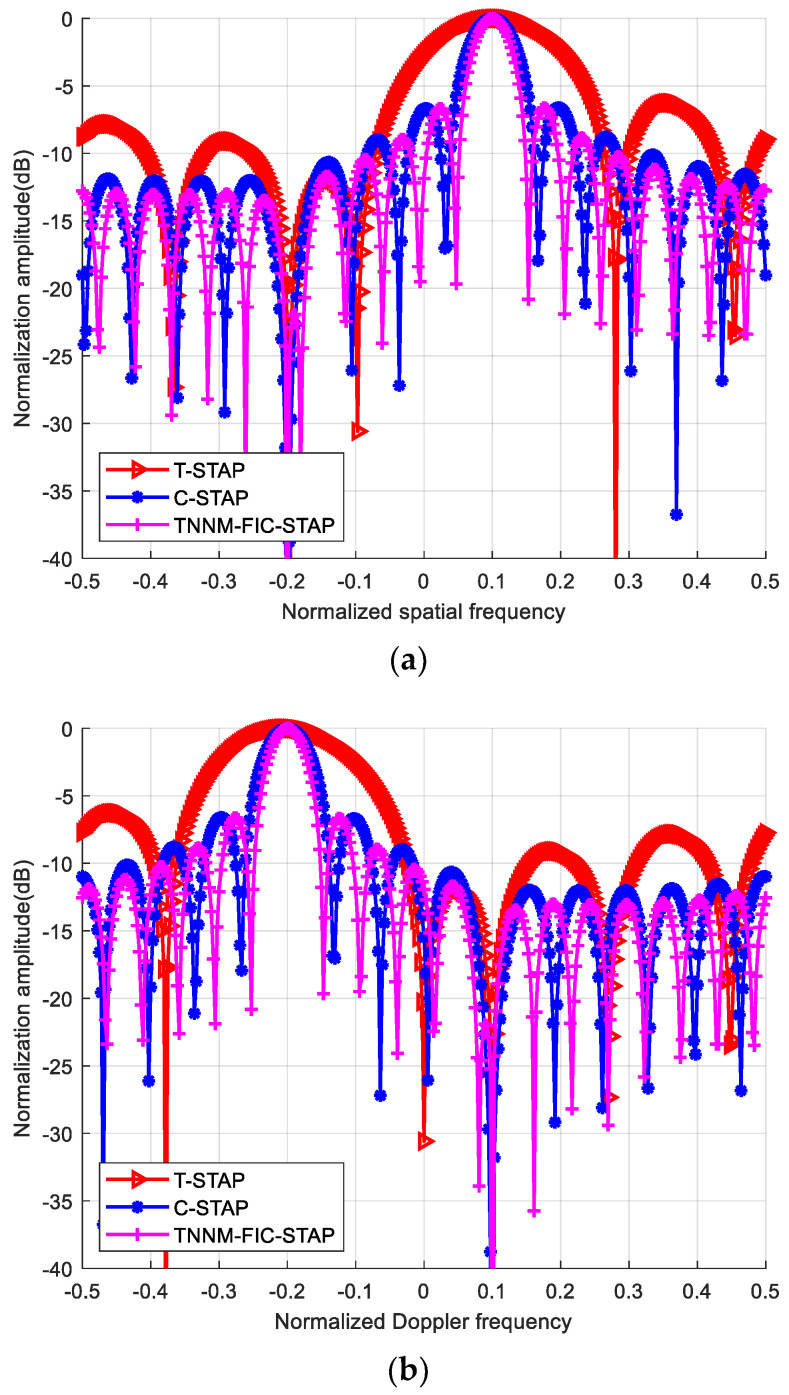
Beampatterns: (**a**) spatial domain; (**b**) Doppler domain.

To better compare the notch-forming capabilities of various algorithms, Figure 6 presents the spatial and temporal channel direction diagrams where the target is located. As can be seen from the figure, the proposed TNNM-FIC-STAP algorithm achieves the optimal spatial and temporal resolution at the target’s position. The closer to the grid boundary, the relatively poorer the resolution becomes.

### 4.4. SCNR

**Figure 7:**
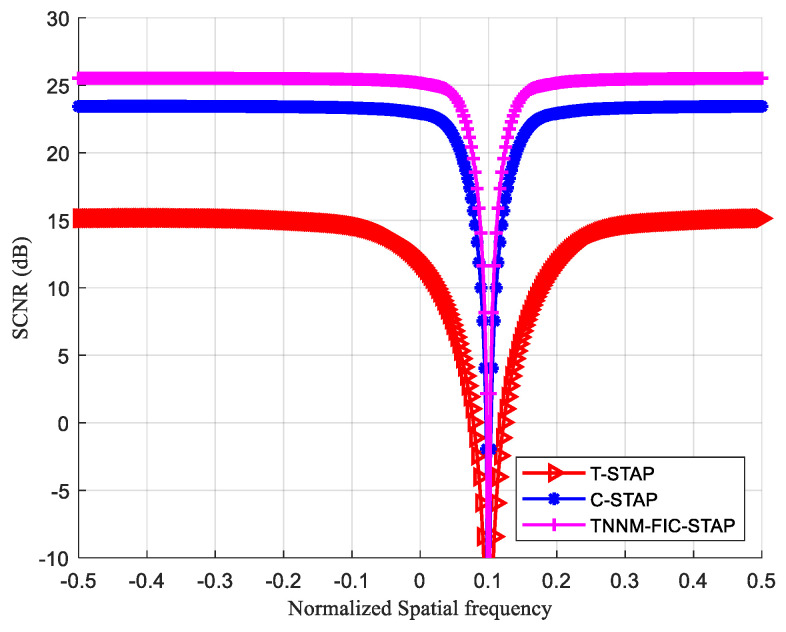
SCNR.

Figure 7 shows that the SCNR performance of the TNNM-FIC-STAP is superior to the T-STAP and C-STAP, indicating that the estimation of CCM is the most accurate. Due to the relatively small number of sensors and pulses in the C-STAP, there is a significant estimation error in the clutter subspace, resulting in a decrease in system performance. Both C-STAP and TNNM-FIC-STAP adopt the coprime structure and difference operation to extend their virtual uniform linear array and virtual uniform linear impulse train, thereby increasing the virtual space DOF and virtual time DOF, resulting in a higher virtual DOF for the system filter. The TNNM-FIC-STAP performs better than C-STAP, because the truncated kernel norm imposes a more stringent constraint on the clutter rank, and its iterative estimation of the CCM leads to smaller errors.

## 5. Conclusions

A fast iterative coprime STAP method based on truncated kernel norm minimization has been proposed. This method uses limited physical array elements and physical pulses, based on difference operations, to generate difference coarrays and copulse, and provides a covariance matrix with missing data. Then, the truncated kernel norm is used to constrain the clutter rank to solve the CCM estimation problem, ensuring the low-rank characteristics of the CCM. The low-rank matrix recovery model is built on the low-rank constraint by truncated kernel norm minimization, which transforms the non-convex optimization problem into a convex one, along with soft-threshold rank estimation of each matrix produced at each iteration. This results in a fast iterative algorithm that does not depend on the matrix rank, which reduces the computational complexity of the algorithm and improves the computational efficiency. The data of the virtual CCM in the difference structure are completed, which improves the system space DOF and time DOF and improves the target temporal resolution and spatial resolution of STAP detection under limited physical array sensors and physical pulses. That is, to meet the scheduled performance of the early warning radar system, the hardware equipment and power loss are relatively reduced, which can effectively increase the UAV equipment and endurance time. While meeting the predetermined performance of the early warning radar system, the hardware equipment and power loss required by the system are relatively reduced, which can effectively increase the unmanned aerial vehicles equipment and battery life. Compared to traditional STAP, the proposed method retains the coprime sampling structure to reduce the mutual coupling effect. Theoretical analysis shows the improved DOF of the system filter under a limited number of N physical sensors and M physical pulses. In simulation experiments, this method has been proven to be able to achieve higher target detection and reduce mutual coupling effects under limited physical array sensors and pulses, which is better than typical STAP methods such as T-STAP and C-STAP. Practical experiments show that the TNNM-FIC-STAP provides more accurate clutter suppression and less environmental influence when detecting dynamic targets. In fact, radar may lose some data. These missing data will affect the robustness of the algorithm, so it is necessary to study their robustness and the percentage of allowed data loss in the next step.

## Figures and Tables

**Table 1:** List of abbreviations.

Abbreviation	Full Name
STAP	space-time adaptive processing
TKNM	truncated kernel norm minimization
CCM	clutter covariance matrix
SINR	signal-to-interference-to-noise ratio
DOF	degrees of freedom
TNNM-FIC-STAP	fast iterative coprime STAP algorithm
PRI	pulse repetition interval
CPI	coherent processing interval
ULAs	uniform linear arrays
CNCM	clutter plus noise covariance matrix
SNR	signal-to-noise ratio
SVD	singular value decomposition
SVT	singular value threshold
T-STAP	traditional STAP
C-STAP	traditional coprime STAP
RMSE	root mean square error
SCM	sample covariance matrix
NNM	minimum nuclear norm

**Table 2:** Computational complexity.

Step	Computational Complexity
Step 1	$O({\left(4N_{1}N_{2}-2N_{2}+1\right)}^{2}\times {\left(4M_{1}M_{2}-2M_{2}+1\right)}^{2})$
Step 2	$O({\left(4N_{1}N_{2}-2N_{2}+1\right)}^{3}\times {\left(4M_{1}M_{2}-2M_{2}+1\right)}^{3})$
Step 3	$O({\left(4N_{1}N_{2}-2N_{2}+1\right)}^{2}\times {\left(4M_{1}M_{2}-2M_{2}+1\right)}^{2})$
Step 4	$O({\left(4N_{1}N_{2}-2N_{2}+1\right)}^{2}\times {\left(4M_{1}M_{2}-2M_{2}+1\right)}^{2})$
Step 5	O(1)

**Table 3:** Main parameters of STAP radar.

Symbol	Quantity	Value
N	the number of sensors	6
M	the number of pulses	6
λ	carrier wavelength	0.2 m
$T_{r}$	minimal PRI	0.5 ms
$v_{r}$	radar velocity	100 m/s
$\sigma_{n}^{2}$	noise power	1 dB
$N_{c}$	number of independent clutter patches	361
CNR	clutter to noise ratio	30 dB
SNR	Signal-to-noise ratio	0 dB
	normalized angle frequency of target	0.1
	normalized Doppler frequency of target	0.2

## Data Availability

Data are contained within the article.
